# Musashi-2, a novel oncoprotein promoting cervical cancer cell growth and invasion, is negatively regulated by p53-induced miR-143 and miR-107 activation

**DOI:** 10.1186/s13046-017-0617-y

**Published:** 2017-10-26

**Authors:** Peixin Dong, Ying Xiong, Sharon J. B. Hanley, Junming Yue, Hidemichi Watari

**Affiliations:** 1 Department of Women’s Health Educational System, Hokkaido University School of Medicine, Hokkaido University, Sapporo, 0608638 Japan; 2 Department of Gynecology, State Key Laboratory of Oncology in South China, Sun Yat-sen University Cancer Center, Guangzhou, 510060 China; 3 Department of Pathology and Laboratory Medicine, University of Tennessee Health Science Center, Memphis, TN 38163 USA; 4 Center for Cancer Research, University of Tennessee Health Science Center, Memphis, TN 38163 USA; 5 Department of Obstetrics and Gynecology, Hokkaido University School of Medicine, Hokkaido University, Sapporo, 0608638 Japan

**Keywords:** Musashi-2, C-FOS, p53, microRNA-143, microRNA-107, Mithramycin a, Anti-tumor antibiotic, Cervical cancer, Metastasis

## Abstract

**Background:**

Although previous studies have shown promise for targeting Musashi RNA-binding protein 2 (MSI-2) in diverse tumors, the role and mechanism of MSI-2 for cervical cancer (CC) progression and the regulation of MSI-2 expression remains unclear.

**Methods:**

Using gene expression and bioinformatic analysis, together with gain- and loss-of-function assays, we identified MSI-2 as a novel oncogenic driver and a poor prognostic marker in CC. We explored the regulation of *c-FOS* by MSI-2 via RNA-immunoprecipitation and luciferase assay, and confirmed a direct inhibition of MSI-2 by miR-143/miR-107 using luciferase assay. We assessed the effect of a natural antibiotic Mithramycin A on p53, miR-143/miR-107 and MSI-2 expression in CC cells.

**Results:**

*MSI-2* mRNA is highly expressed in CC tissues and its overexpression correlates with lower overall survival. MSI-2 promotes CC cell growth, invasiveness and sphere formation through directly binding to *c-FOS* mRNA and by increasing c-FOS protein expression. Furthermore, miR-143/miR-107 are two tumor suppressor miRNAs that directly bind and inhibit MSI-2 expression in CC cells, and downregulation of miR-143/miR-107 associates with poor patient prognosis. Importantly, we found that p53 decreases the expression of MSI-2 through elevating miR-143/miR-107 levels, and treatment with a natural antibiotic Mithramycin A increased p53 and miR-143/miR-107 expression and reduced MSI-2 expression, resulting in the inhibition of CC cell proliferation, invasion and sphere formation.

**Conclusions:**

These results suggest that MSI-2 plays a crucial role in promoting the aggressive phenotypes of CC cells, and restoration of miR-143/miR-107 by Mithramycin A via activation of p53 may represent a novel therapeutic approach for CC.

## Background

Cervical cancer (CC) is the fourth most common cause of cancer death in women worldwide [1]. Of all CC patients, approximately 70%–80% were squamous cell carcinoma and the other 10%–15% were adenocarcinomas [2]. More than 70% of CC cases can be attributed to two types of human papillomavirus (HPV) (HPV-16 and HPV-18) [2]. In addition, alterations of the PTEN/PI3K/AKT pathway and overexpression of c-FOS have been implicated in cervical tumorigenesis and progression [3–7]. The abrogation of tumor suppressor protein p53 is responsible for increased aggressiveness of CC [8]. Moreover, the crucial roles for microRNAs (miRNAs) in CC metastasis have been reported [9, 10]. miR-143 and miR-107 are p53-responsive miRNAs [11, 12] and function as tumor suppressors in CC [13–15]. Musashi RNA-binding protein 2 (MSI-2) was proposed to be a potential oncoprotein regulating cancer initiation, progression and drug resistance in leukemia and several solid tumors [16]. However, the role and mechanism of MSI-2 for CC progression and the regulation of MSI-2 expression is poorly understood.

Mithramycin A is a DNA-binding, anti-tumor antibiotic originally isolated from Streptomyces strains [17–19]. Mithramycin A was well-tolerated and effectively reduced tumor growth in mouse xenograft models of CC [20]. Recent evidences have revealed that the anti-cancer effects of Mithramycin A rely on the activation p53 pathway [21]. Currently, no data have been reported concerning its impact on p53 and MSI-2 expression and metastasis-associated properties in CC cells, although Mithramycin A was shown to repress the expression of MSI-2 in lung cancer [22].

c-FOS, a major subunit of the transcription factor activator protein (AP)-1, has been identified in human cancers as a proto-oncogene, which controls cancer cell growth and invasion [23]. Increased expression of c-FOS was associated with high-grade diseases and poor outcome in osteosarcoma and endometrial cancer [24, 25]. c-FOS protein expression was significantly higher in invasive CC than in precancerous lesions of the cervix [26].

In this study, we show that upregulation of MSI-2 cause increase in the expression of c-FOS, resulting in the promotion of CC cell invasion, proliferation and sphere formation. Furthermore, treatment with Mithramycin A restored the expression of miR-143 and miR-107 (two direct suppressors of MSI-2) via activation of p53, leading to inhibition of MSI-2 expression and reduced proliferation, invasion and sphere formation of CC cells.

## Methods

### Cell culture and reagents

Human CC cell lines HeLa and SiHa (American Type Culture Collection, Manassas, VA) and immortalized normal cervical epithelial squamous cell line H8 (Chinese Academy of Sciences Cell Bank, Shanghai, China) were cultured in DMEM/F12 medium (Invitrogen) supplemented with 10% fetal bovine serum (Invitrogen). These cell lines were routinely tested by PCR for mycoplasma contamination by using the following primers: Myco_fw1: 5′-ACACCATGGGAGCTGGTAAT-3′, Myco_rev1: 5′-CTTCATCGACTTTCAGACCCAAGGCA-3′. MiRNA mimic and miRNA inhibitor for miR-143 or miR-107, and respective controls were obtained from Ambion (Austin, TX). MSI-2 small interfering RNA (siRNA) and control siRNA were obtained from Santa Cruz Biotechnology (Santa Cruz, CA). The cDNA plasmid encoding human MSI-2, c-FOS and p53 were purchased from OriGene (Rockvill, MD). Transient transfection experiments were performed using Lipofectamine 3000 (Invitrogen, Carlsbad, CA) according to the manufacturer’s protocol. Mithramycin A was obtained from (Sigma-Aldrich, St. Louis, MO).

### Real-time reverse transcription-PCR (qRT-PCR)

The total RNA was isolated using TRIzol (Invitrogen, Carlsbad, CA) according to the manufacturer’s instructions. 100 ng of total RNA from each sample was subjected to first-strand cDNA synthesis using PrimeScript RT reagent kit (Takara, Otsu, Japan). For mature miRNA quantitation, miR-143 or miR-107 expression was determined using the NCode miRNA qRT**-**PCR analysis (Invitrogen, Carlsbad, CA) following manufacturer-recommended protocols. Forward primer is the exact sequence of the mature miR-143 or miR-107. The primers used in the PCR reaction were purchased from Applied Biosystems (Carlsbad, CA). Real-time PCR was conducted by using ABI PRISM 7000 Sequence Detection System (Applied Biosystems). *GAPDH* or U6 was used as internal control for the normalization of mRNA or miRNA, respectively. qRT-PCRs were performed in triplicate, and data was presented as fold change from control.

### Western blot analysis

Total proteins were extracted from cell lines 48 h after transfections using M-PER reagent (Pierce, Rockford, IL). The protein concentration was determined using the Bio-Rad protein assay system (Bio-Rad, Hercules, CA). Total protein lysates (30 μg) were fractionated using SDS-PAGE and transferred onto PVDF membranes. The following primary antibodies were used: anti-MSI-2 (Abcam, ab76148), anti-c-FOS (Abcam, ab156802), anti-PTEN (Abcam, ab32199), anti-p53 (Santa Cruz, sc-126), anti-p21 (Santa Cruz, sc-6246) and anti-GAPDH (Santa Cruz, sc-47,778). Blots were developed using horseradish peroxidase-conjugated secondary antibody and the enhanced chemiluminescence detection system (Amersham, Little Chalfont, UK). Primary and secondary antibodies were used at 1:1000 and 1:5000 dilutions, respectively.

### Transwell invasion assay

The invasive ability of CC cells was tested using BioCoat Matrigel Invasion Chamber (BD Biosciences, San Jose, CA) as described previously [27, 28]. Briefly, transfected CC cells with serum-free medium were seeded into the upper chamber of the system. Bottom wells in the system were filled with complete medium. After 24 h of incubation, the cells in the upper chamber were gently removed with a cotton swab, and the cells that invaded through Matrigel matrix membrane were stained with Giemsa. Then, the number of invaded cells were counted under a microscope.

### Cell proliferation assay

Cell proliferation was measured by using cell counting kit-8 (CCK-8) following manufacturer’s instruction (Dojindo, Kumamoto, Japan). CC cells were plated in 96-well plates at a density of 1 × 10^4^ cells per well and subjected to the indicated transfection or treatment. After 72 h of incubation, 10 μl of CCK-8 solution was added into each well and the plates were incubated for additional 4 h at 37 °C. The absorbance at 450 nm was determined using a microplate reader. The experiment was performed in triplicate wells and repeated three times.

### Sphere formation assay

Single-cell suspensions were suspended at a density of 5000 cells/ml in serum-free DMEM/F12 medium containing N2 plus media supplement (Invitrogen, CA), epidermal growth factor (20 ng/ml), basic fibroblast growth factor (20 ng/ml) and heparin (4 mg/ml) in 6-well Ultra-Low attachment plates (Corning, NY). Fresh medium was added to each well every 3 days. The suspension cultures were continued for 14 days, and then the number of spheres larger than 50 μm was counted.

### RNA immunoprecipitation

RNA immunoprecipitation (RNA-IP) was performed using Magna RIP RNA Binding Protein Immunoprecipitation Kit (Millipore, Billerica, MA) according with manufacturer’s instructions. In brief, cells were washed with cold phosphate-buffered saline and lysed with RIP lysis buffer provided in the kit. Next, 5 μg of anti-MSI-2 antibody (part of the kit 03–115; Millipore) or anti-IgG control antibody (Millipore) was incubated with magnetic beads, and used to immunoprecipitate endogenous MSI-2-RNA complexes. After the immunoprecipitated complexes were washed, they were treated with proteinase K. RNA extraction was performed by the phenol–chloroform method, and purified RNA was used for qRT–PCR to check RNA binding with MSI-2 protein. Results are presented relative to IgG immunoprecipitation, set as 1.

### Luciferase reporter assay

Human *c-FOS* or *MSI-2* 3′-UTR luciferase-reporter vector was obtained from OriGene Technologies (Rockville, MD). The mutant *c-FOS* 3′-UTR vector containing the mutation in predicted MSI-2-binding sequence (TAGTA to AAAAA), or the mutant *MSI-2* 3′-UTR vector carrying mutations at putative miR-143 or miR-107-binding site, were generated using a QuickChange site-directed mutagenesis kit (Stratagene, CA). CC cells were transfected with firefly luciferase reporter vector, Renilla reporter plasmid pRL-CMV, together with MSI-2 siRNA, miRNA mimic, anti-miRNA inhibitor or their negative controls. 24 h after transfection luciferase activity was assessed with the Dual-Luciferase Reporter Assay system (Promega). The ratio of firefly/Renilla luciferase activity was determined and reported as relative luciferase activity. The relative luciferase activity in cells transfected with control siRNA, control miRNA mimic or control miRNA inhibitor was set to 1.

### Clinical samples

Following an institutional review board-approved protocol, primary CC specimens (*n* = 58) and normal tumor-adjacent cervical tissues (*n* = 58) were collected at the Cancer Center, Sun Yat-Sen University, China. All samples were obtained at primary resection, and none of the patients had been subjected to chemotherapy or radiation therapy before resection. Samples were snap-frozen and stored in liquid nitrogen until RNA extraction.

### Statistical analysis

The results are presented as the mean ± SEMs from at least three independent replicates. For experiments in vitro, 2-tailed Student’s *t*-test or 1-way ANOVA was used. The difference in mRNA or miRNA expression between CC and normal cervical tissues were evaluated using the nonparametric Mann-Whitney *U*-Test. *P*-values <0.05 were regarded as significant.

## Results

### *MSI-2* is overexpressed in human CC tissues and correlates with poor patient survival

**Fig. 1:**
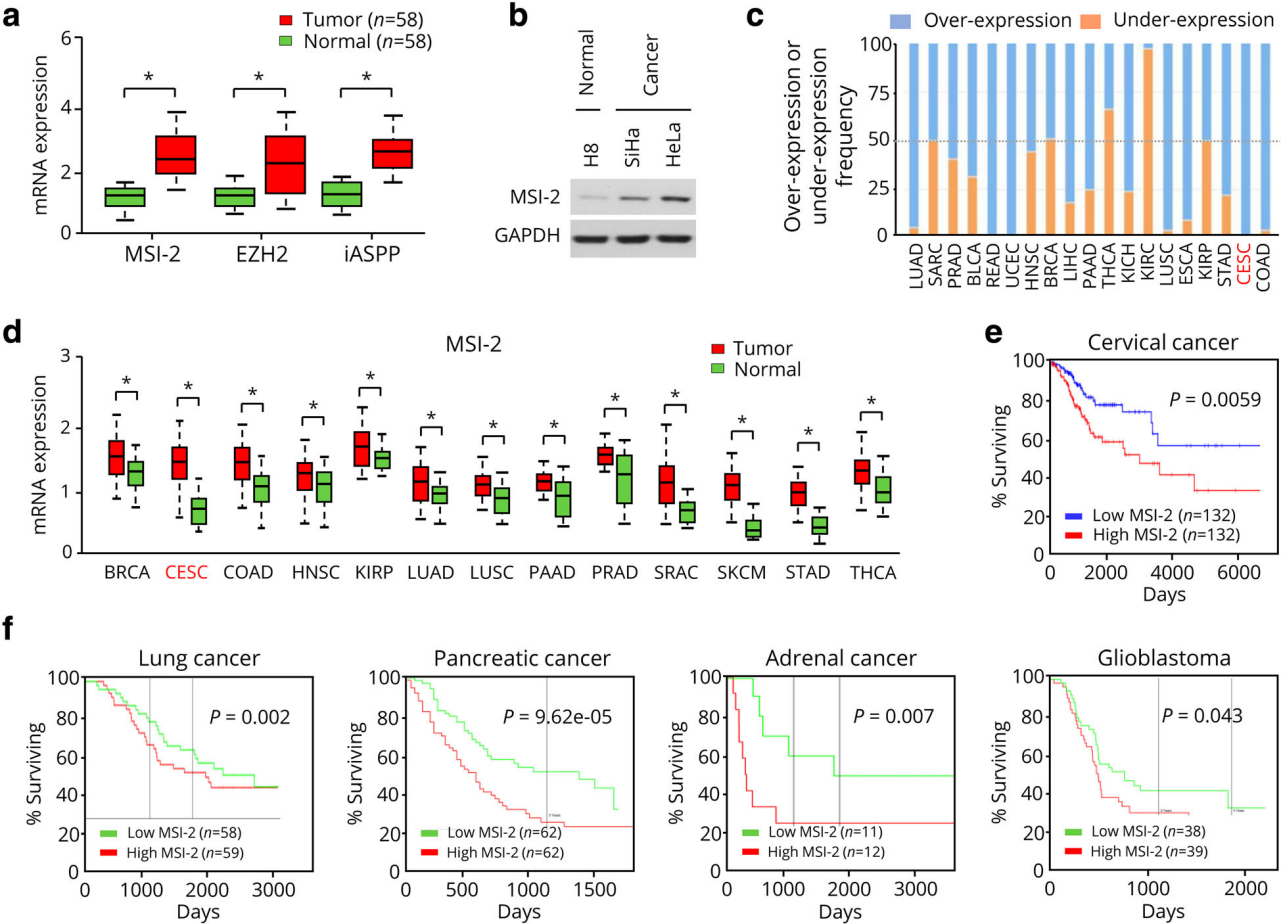
Musashi-2 (MSI-2) is overexpressed in cervical cancer tissues and correlates with poor patient survival. **a,** Relative mRNA expression of *MSI-2*, *EZH2* and *iASPP* in 58 matched human normal cervix tissues and cervical cancer (CC) tissues. **b,** Western blotting analysis of MSI-2 protein expression in CC cell lines and normal cervical cell line H8. **c,** Pan-cancer analysis of *MSI-2* mRNA expression in a variety of human cancerous tissues including cervical (CESC), prostate (PRAD), bladder (BLCA), rectum (READ), endometrial (UCEC), liver (LIHC), pancreatic (PAAD), lung squamous (LUSC), esophageal (ESCA), stomach (STAD), and colon (COAD) cancer relative to their paired normal tissues using the BioExpress database. **d,** Analysis of *MSI-2* mRNA expression in diverse cancer types including cervical, breast (BRCA), head and neck (HNSC), kidney papillary (KIRP), lung adenocarcinoma (LUAD), sarcoma (SARC), skin melanoma (SKCM), and thyroid (THCA) cancer relative to the corresponding normal tissues (MethHC database). **e,** Kaplan–Meier overall survival curves for 264 CC patients stratified by median *MSI-2* mRNA expression (OncoLnc database). **f,** Kaplan-Meier plots overall survival curves for lung, pancreatic, adrenal and glioblastoma patients stratified by median *MSI-2* mRNA expression (PROGgene database). ^*^ *P* < 0.05

Using qRT-PCR analysis, we observed a significantly increased expression of *MSI-2* as well as two known oncogenes (*EZH2* and *iASPP*) [9, 28] in CC tissues compared with normal cervical tissues (Fig. 1a). Consistently, HeLa and SiHa human CC cells showed abundant MSI-2 protein expression, whereas this protein had weak expression in immortalized human cervix epithelial cell line H8 (Fig. 1b). By using the BioExpress database [29], we analyzed the TCGA pan-cancer data to evaluate *MSI-2* mRNA expression in a variety of human normal and cancerous tissues based on the Affymetrix GeneChip platform. Compared to normal tissues, *MSI-2* expression was elevated in numerous human cancers including cervical, prostate, bladder, rectum, endometrial, liver, pancreatic, lung, esophageal, stomach and colon cancer (Fig. 1c). Moreover, we used the MethHC database that provided gene expression patterns from TCGA across 18 major human cancers [30] to compare the mRNA expression of *MSI-2* in normal and tumor tissues. The *MSI-2* mRNA expression was highly overexpressed in a variety of cancer types, including cervical, breast, head and neck, kidney papillary, lung, sarcoma, skin melanoma, and thyroid cancer as compared to the corresponding normal tissues (Fig. 1d). Furthermore, we retrieved cancer data sets from the OncoLnc web tool [31] that links TCGA survival data of CC patients to mRNAs to determine the prognostic value of MSI-2 in human CC. 264 CC patients were divided into high expression group and low expression group by the median. Higher expression of *MSI-2* was significantly correlated with worse disease prognosis (Fig. 1e). We also defined the relationship between *MSI-2* mRNA expression and the overall survival in human lung, pancreatic, adrenal cancer and glioblastoma patient data sets using the PROGgene web site [32]. For these tumor types, high expression of *MSI-2* mRNA was significantly associated with poor prognosis (Fig. 1f). Taken together, these results indicate that MSI-2 may function as a tumor promoter in CC and possibly other tumor types.

### MSI-2 overexpression promotes invasion, proliferation and sphere formation of CC cells

**Fig. 2:**
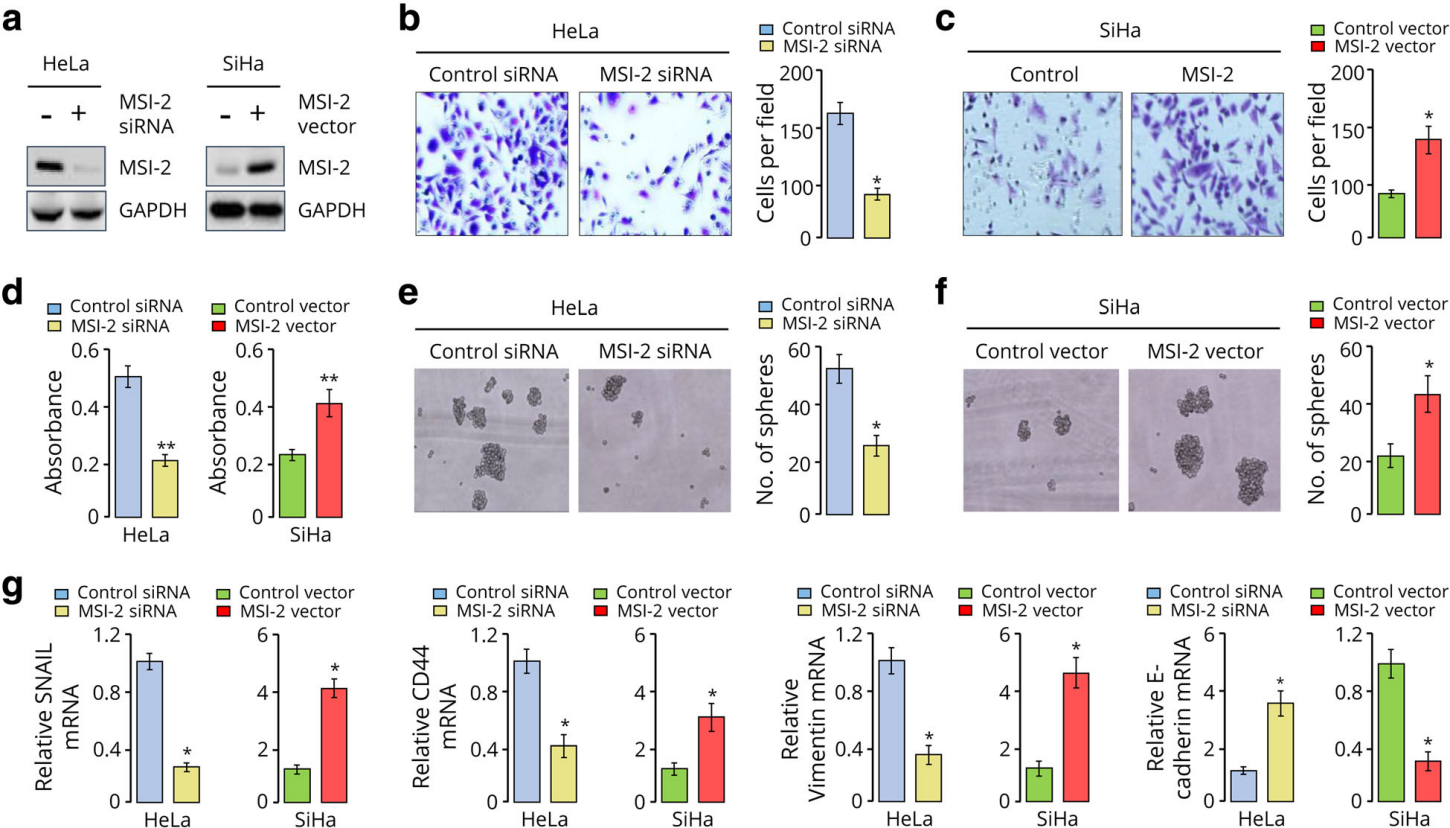
MSI-2 overexpression promotes invasion, proliferation and sphere formation of CC cells. **a,** Western blotting analysis of MSI-2 in indicated cells. GAPDH served as the loading control. **b** and **c,** Representative images (left) and quantification (right) of invaded Hela cells (**b**) and SiHa cells (**c**), as analyzed using Matrigel invasion assay. **d,** Cell proliferation was measured using a cell counting kit-8 assay. **e** and **f,** Representative images (left) and quantification (right) of sphere formed from HeLa (**e**) and SiHa (**f**) cells. **g**, qRT-PCR analysis of *SNAIL*, *Vimentin*, *CD44* and *E-cadherin* in HeLa cells. ^*^ *P* < 0.05

To enable functional characterization of MSI-2, we transiently transfected HeLa cells that express relatively higher levels of MSI-2 protein with either human MSI-2 siRNA or control siRNA. In addition, SiHa cells that show relatively lower levels of MSI-2 were transfected with control plasmid alone or with expression plasmid coding for *MSI-2*. Downregulation or up-regulation of MSI-2 protein was confirmed using Western blotting analysis (Fig. 2a). We used Matrigel invasion chamber assay to examine the invasive potential of CC cells transfected with specific MSI-2 siRNA or MSI-2 expression plasmid. In contrast to HeLa cells transfected with control siRNA, MSI-2 siRNA-transfected HeLa cells showed a significantly lower level of penetration (Fig. 2b). However, the number of cells passed across the Matrigel layer was significantly increased in SiHa cells transduced with MSI-2 expression plasmid, compared to control plasmid (Fig. 2c). These results indicated that overexpression of MSI-2 can enhance in vitro tumor invasion for CC cells.

To evaluate the effects of MSI-2 modulation on CC cell proliferation, we performed CCK-8 assay, and found that MSI-2-depleted HeLa cells had significantly reduced growth rates, and MSI-2 overexpression caused a significant increase in the cell growth rate (Fig. 2d). The contribution of MSI-2 to cancer stemness was then assessed via sphere formation assay. The number of sphere was significantly decreased in MSI-2-depleted HeLa cells, buy significantly increased in SiHa cells overexpressing MSI-2 (Fig. 2e and f), indicating that MSI-2 has a critical function in CC cell growth.

To elucidate the molecular basis whereby MSI-2 promotes the malignant behaviors of CC cells, we examined the mRNA expression of several metastasis-related genes (*SNAIL*, *Vimentin* and *E-cadherin*) and cancer stem cell marker *CD44* in MSI-2 overexpression cells or knockdown cells. The results showed that the levels of *SNAIL*, *Vimentin* and *CD44* were decreased in MSI-2 knockdown HeLa cells. Conversely, inhibition of MSI-2 induced the mRNA levels of *E-cadherin* (Fig. 2g). The opposite results were found in SiHa cells when MSI-2 was overexpressed (Fig. 2g). These results point to the crucial role of MSI-2 activation in accelerating CC cell invasion and proliferation.

### MSI-2 regulates CC cell invasion and growth through translational control of c-FOS

**Fig. 3:**
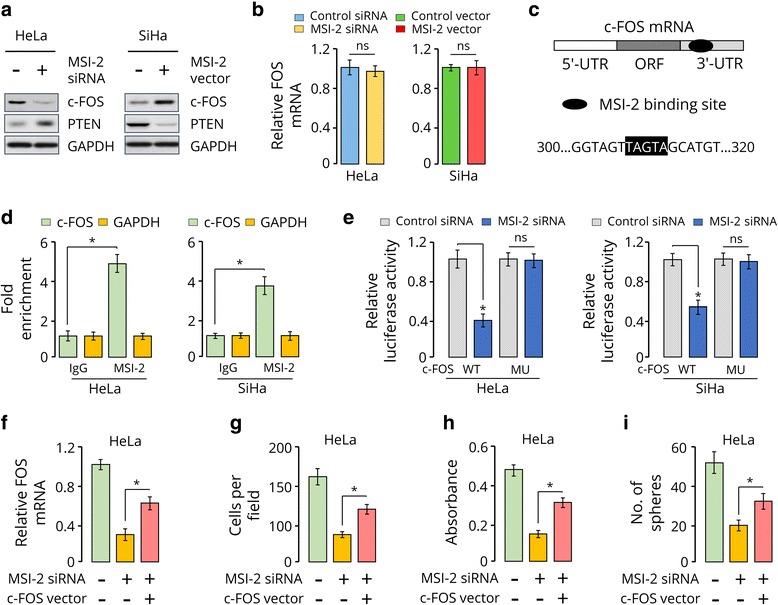
MSI-2 regulates CC cell invasion and growth through translational control of c-FOS. **a**, Western blotting analysis of c-FOS and PTEN in indicated cells. GAPDH served as the loading control. **b**, qRT-PCR analysis of c-*FOS* expression in indicated cells. **c**, Predicted MSI-2 binding site in the 3′-UTR of c-*FOS* mRNA. **d**, RNA-immunoprecipitation in CC cells using an anti-IgG control or anti-MSI-2 antibody. c-*FOS* or *GAPDH* mRNA abundance in immune-precipitated fraction was determined by qRT-PCR. Results are presented relative to IgG immunoprecipitation, set as 1. **e**, The c-*FOS* 3′-UTR reporter constructs containing the wild-type (WT) or mutant (MU) MSI-2 binding sequences were co-transfected with control siRNA or MSI-2 siRNA into CC cells and luciferase activity levels measured. ns, not significant. **f**, qRT-PCR analysis of *c-FOS* mRNA in HeLa cells transfected as indicated. **g**, **h** and **i**, Quantification of invasion (**g**), proliferation (**h**) and sphere formation (**i**) of HeLa cells transfected as indicated. ^*^ *P* < 0.05

In pancreatic cancer cells, MSI-2 loss led to down-regulation of many key proto-oncogenes including c-FOS [33]. Because c-FOS plays a key role in cancer progression of various cancer types, including CC [7, 34], we examined whether its expression is regulated by MSI-2 in CC cells. Immunoblotting analysis showed that the levels of c-FOS significantly declined in MSI-2-depleted HeLa cells. However, c-FOS protein expression was increased following MSI-2 overexpression in SiHa cells (Fig. 3a). As a control, the expression of tumor suppressor PTEN, a downstream target of MSI-2 [35], was examined. We found that knockdown of MSI-2 increased PTEN levels, whereas overexpression of MSI-2 reduced its expression (Fig. 3a). qRT-PCR analysis suggested that the levels of *c-FOS* mRNA was not affected following either knockdown or overexpression of MSI-2 (Fig. 3b), suggesting that MSI-2 may induce c-FOS protein levels by controlling *c-FOS* mRNA translation.

We identified one MSI-2 binding site (TAGTA) in the *c-FOS* 3′-UTR region (Fig. 3c) [36, 37]. Using RNA-IP, we assessed whether MSI-2 protein binds to *c*-*FOS* mRNA in CC cells. The results showed that the mRNA of *c-*FOS was highly enriched in MSI-2-antibody precipitated RNA fraction in HeLa and SiHa cells (Fig. 3d). To examine the direct interaction between of MSI-2 protein and c-*FOS* mRNA, c-FOS 3′-UTR reporter construct carrying the wild-type or the mutant MSI-2 binding site was tested. Compared with the control siRNA, we detect a significant decrease in luciferase activity of wild-type *c-FOS* 3′-UTR reporter when we transfected MSI-2 siRNA in HeLa and SiHa cells (Fig. 3e). However, down-regulation of MSI-2 had no repressive effect on the mutant *c-FOS* 3′-UTR construct (Fig. 3e). In total, our experiments indicated that MSI-2 facilitates *c-FOS* mRNA translation, via direct binding to its 3′-UTR. We further investigated whether c-FOS is indeed involved in MSI-2-mediated tumor promotion in CC. A plasmid expressing c-*FOS* cDNA was co-transfected with MSI-2 siRNA in HeLa cells (Fig. 3f). Importantly, overexpression of *c-FOS* partially rescued MSI-2 siRNA-inhibited cell invasion, proliferation and sphere formation (Fig. 3g–i). These results support that MSI-2 promotes CC cell growth, invasiveness and sphere formation by facilitating *c*-*FOS* mRNA translation.

### miR-143 and miR-107 target MSI-2 and inhibit CC cell proliferation and invasion

**Fig. 4:**
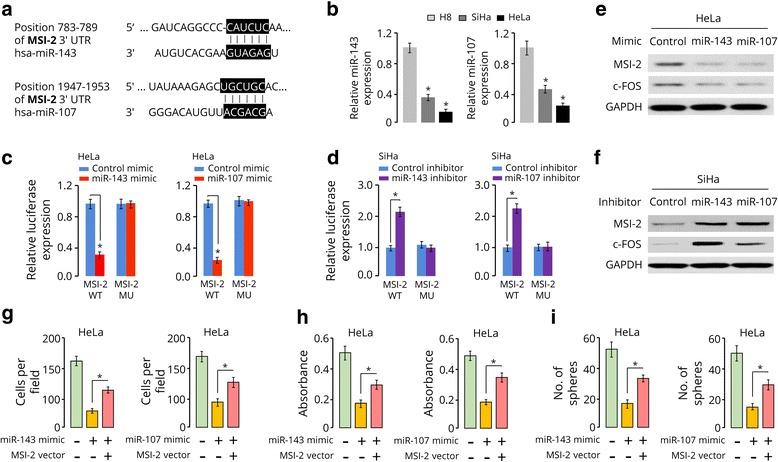
miR-143 and miR-107 target MSI-2 and inhibit CC cell proliferation and invasion. **a,** Predicted miR-143 and miR-107 binding sites in the 3′-UTR of *MSI-2*. **b**, miR-143 and miR-107 expression in normal cell line H8 and CC cell lines (HeLa and SiHa). **c** and **d**, Luciferase activities of reporter construct containing the wild-type (WT) or mutant (MU) 3′-UTR of *MSI-2* in HeLa (**c**) and SiHa (**d**) cells transfected as indicated. **e** and **f**, Western blot analysis of HeLa (**e**) and SiHa (**f**) cells transfected as indicated. **g**, **h** and **i**, Quantification of invasion (**g**), proliferation (**h**) and sphere formation (**i**) of HeLa cells transfected as indicated. ^*^ *P* < 0.05

Analysis using publicly available algorithms predicted that miR-143 and miR-107 might be potential regulators of MSI-2 (Fig. 4a). Our qRT-PCR analysis showed that miR-143 and miR-107 expression were significantly reduced in HeLa and SiHa cells compared with normal H8 cells (Fig. 4b). Meanwhile, luciferase assays showed overexpressing miR-143 or miR-107 via miRNA mimic attenuated, whereas silencing of these two miRNAs via miRNA inhibitor elevated the reporter activities driven by the 3′-UTR of MSI-2 transcript (Fig. 4c and d). As expected, the observed luciferase activity changes were abrogated when the miR-143 or miR-107 binding site in MSI-2 3′-UTR was mutated (Fig. 4c and d). Consistently, Western blotting analysis revealed that the expression of MSI-2 as well as c-FOS was dramatically decreased in miR-143 or miR-107 mimic-transfected HeLa cells (Fig. 4e), but increased in miR-143 or miR-107-silenced SiHa cells (Fig. 4f). Moreover, overexpression of MSI-2 significantly reversed miR-143 or miR-107-mediated inhibition of invasion, proliferation and sphere formation of HeLa cells (Fig. 4g–i). These results suggest that miR-143 and miR-107 are direct suppressors of MSI-2 and inhibit CC cell invasion and proliferation.

**Fig. 5:**
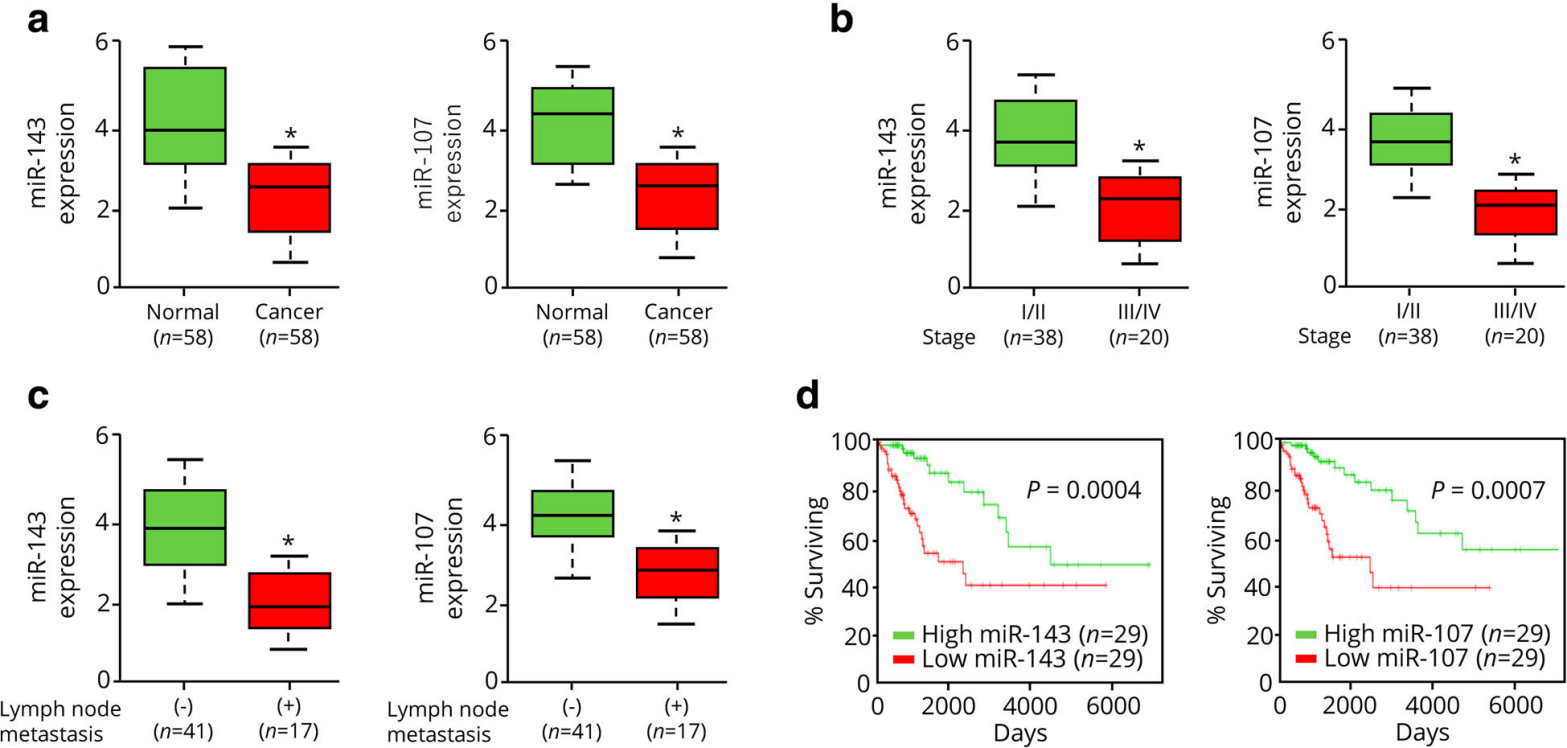
Reduced miR-143 and miR-107 expression correlates with CC progression. **a**, miR-143 and miR-107 expression in adjacent normal cervical tissues and CC tissues. **b**, miR-143 and miR-107 expression in CC tissues at clinical stages I-IV. **c**, miR-143 and miR-107 expression in CC patients with or without lymph node metastasis. **d**, Kaplan–Meier overall survival curves for CC patients stratified by median miR-143 or miR-107 expression. NS, not significant. ^*^ *P* < 0.05

To address the relevance of miR-143 or miR-107 expression to human CC, we examined the levels of these miRNAs in primary CCs using qRT-PCR analysis. Both miR-143 and miR-107 were markedly downregulated in cancer tissues as compared with adjacent normal cervical tissues (Fig. 5a). The qRT-PCR analysis also suggested that miR-143 and miR-107 expression levels were significantly lower in CC patients with advanced stage disease (Fig. 5b), and in CC patients with lymph node metastasis (Fig. 5c). Notably, we found that patients with lower expression of miR-143 or miR-107 had a shorter survival time (Fig. 5d). Taken together, these results suggest that reduced miR-143 and miR-107 expression correlates with CC progression.

### Upregulation of miR-143 and miR-107 by Mithramycin a via p53 activation decreases MSI-2 levels and suppresses invasion and proliferation of CC cells

**Fig. 6:**
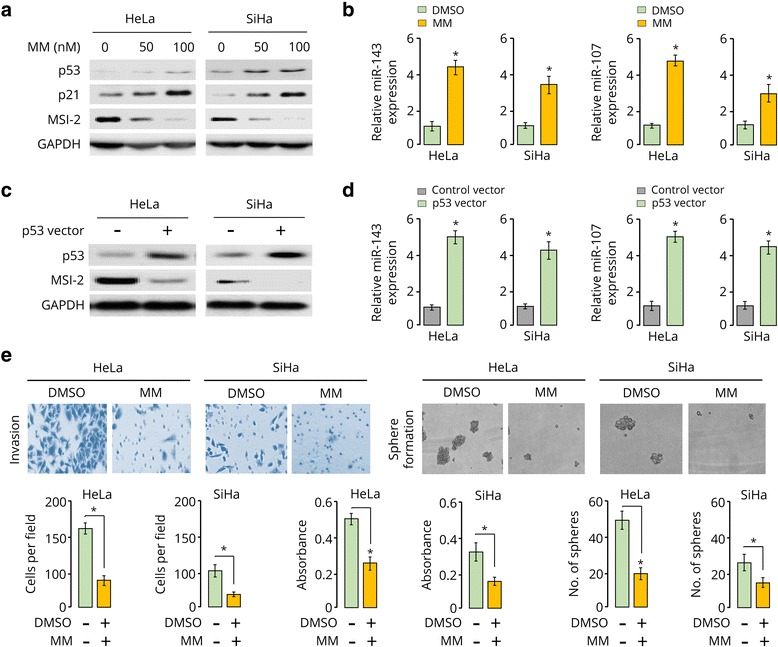
Upregulation of miR-143 and miR-107 by Mithramycin A via p53 activation decreases MSI-2 levels and suppresses invasion and proliferation of CC cells. **a**, Western blotting analysis of p53, p21 and MSI-2 in CC cells treated with Mithramycin A (MM). **b,** miR-143 and miR-107 expression in CC cells treated with Mithramycin A. **c**, Western blotting analysis of p53 and MSI-2 in CC cells transfected with control or p53 expression vector. **d**, qRT-PCR analysis of miR-143 and miR-107 in CC cells transfected with control or p53 expression vector. **e**, Representative images of transwell invasion assays and sphere formation assays (upper panel). Quantification of invasion, proliferation and sphere formation of CC cells treated with Mithramycin A or DMSO (lower panel). ^*^ *P* < 0.05

miR-143 and miR-107 were shown to be induced by tumor suppressor p53 at post-transcriptional and transcriptional levels, respectively [11, 12]. Interestingly, treatment with Mithramycin A led to activation of p53, and can trigger senescence and apoptosis of malignant pleural mesothelioma cells [21]. Therefore, we postulated that the addition of Mithramycin A could increase miR-143 or miR-107 expression through p53 induction, thereby resulting in the downregulation of MSI-2 and the suppression of CC cell invasion and proliferation. Western blot analysis showed that Mithramycin A upregulated the protein expression of p53, p21 (a downstream effector of p53), but downregulated MSI-2 expression in both CC cell lines in a dose-dependent manner (Fig. 6a). Consistently, treatment with Mithramycin A significantly restored the expression of miR-143 and miR-107 in CC cells (Fig. 6b). To understand whether p53 regulates the expression MSI-2, miR-143 and miR-107 in CC cells used in this study, we investigated the impact of transient p53 overexpression on MSI-2 expression. Compared to cells transfected with a control plasmid, HeLa and SiHa cells transfected with a plasmid expressing *p53* cDNA showed reduced protein levels of MSI-2 (Fig. 6c), and exhibited increased expression of miR-143 and miR-107 (Fig. 6d). Finally, cell functional studies demonstrated that Mithramycin A-treated CC cells displayed a reduced capacity of invasion, proliferation and sphere formation than DMSO-treated control cells (Fig. 6e). Collectively, these results suggest that upregulation of miR-143 and miR-107 by Mithramycin A via p53 activation decreases MSI-2 levels and suppresses invasion and proliferation of CC cells.

## Discussion

CC is one of the most common cancers among women worldwide. A better understanding of the molecular mechanisms involved in CC progression is urgently required. MSI-2 plays important roles in contributing to epithelial-mesenchymal transition, migration, invasion, proliferation, cancer stemness and chemoresistance in a variety of human cancer types [16]. Numerous studies have reported that MSI-2 protein is frequently elevated in tumors, including brain, breast, pancreas, colon, lung, ovary and bladder cancer [16], and its overexpression were closely associated with aggressive characteristics and poor prognosis for patients with pancreatic cancer and chronic myeloid leukemia [38, 39]. MSI-2 was found to promote breast cancer progression through binding to estrogen receptor 1 mRNA and inducing its expression [36]. In addition, MSI-2 stimulates migration and invasion of bladder cancers by activating the JAK2/STAT3 signaling pathway [40], and indirectly downregulates tight junction–associated claudins to increase lung cancer cell invasion and metastasis [41]. However, neither the cellular role nor the downstream MSI-2-regulated genes in CC have been reported. Herein, we found that the overexpression of MSI-2 significantly increase the protein expression of key oncoprotein c-FOS through direct interaction with the 3′-UTR of *c-FOS* mRNA and facilitating its translation. Consistently, overexpression of MSI-2 promoted, but downregulation of MSI-2, inhibited invasion, proliferation and sphere formation in CC cells. Therefore, our results provide new insights into the important roles of MSI-2 induction in the activation of c-FOS signaling pathway and promotion of CC progression.

Several mechanisms that influence MSI-2 expression have been reported [35, 42]. The loss of tumor suppressor APC resulted in the activation of MSI-2 in colorectal cancer [35], and reduced expression KLF4 (a transcriptional repressor of MSI-2) led to MSI-2 overexpression in pancreatic cancer [42]. Accumulating evidence indicated that miRNAs are important regulators involved in cancer biology. A previous study indicated a potential role of miR-145 in regulating MSI-2 expression in human endometriotic cells [43]. However, it remains unclear whether the dysregulation of miRNAs accounts for the dysregulation of MSI-2 in CC. Decreased miR-143 expression was detected in CC tissues and introduction of miR-143 suppressed tumor formation in CC cells through suppressing Bcl-2 expression [13]. Moreover, miR-107 inhibited CC cell invasion by targeting MCL-1 [15]. In the present study, miR-143 and miR-107 were suggested to directly suppress MSI-2 expression, leading to inhibition of CC cell invasion, proliferation and sphere formation. Therefore, our results uncover a new regulatory mechanism of MSI-2 activation in CC, and suggest that inhibition of MSI-2 via the restoration of miR-143 and miR-107 might serve as a potential therapeutic target for CC treatment.

miR-143 and miR-107 have been identified as mediators of tumor suppression exerted by the p53 tumor suppressor [11, 12]. Mithramycin A was shown to inhibit the growth of various cancers (including CC) by decreasing Sp1 protein [20, 44, 45]. Importantly, p53 signaling was observed to be a top pathway induced by Mithramycin A in vitro and in vivo [21]. Our results revealed that the upregulation of miR-143 and miR-107 via p53 activation is a key mechanism of Mithramycin A-mediated CC suppression. It has been reported that prolonged Mithramycin A treatment was well tolerated after systemic administration to mice carrying CC cells [20]. Thus, our results provide additional evidence that support the use of Mithramycin A as an effective therapeutic strategy for CC.

## Conclusions

**Fig. 7:**
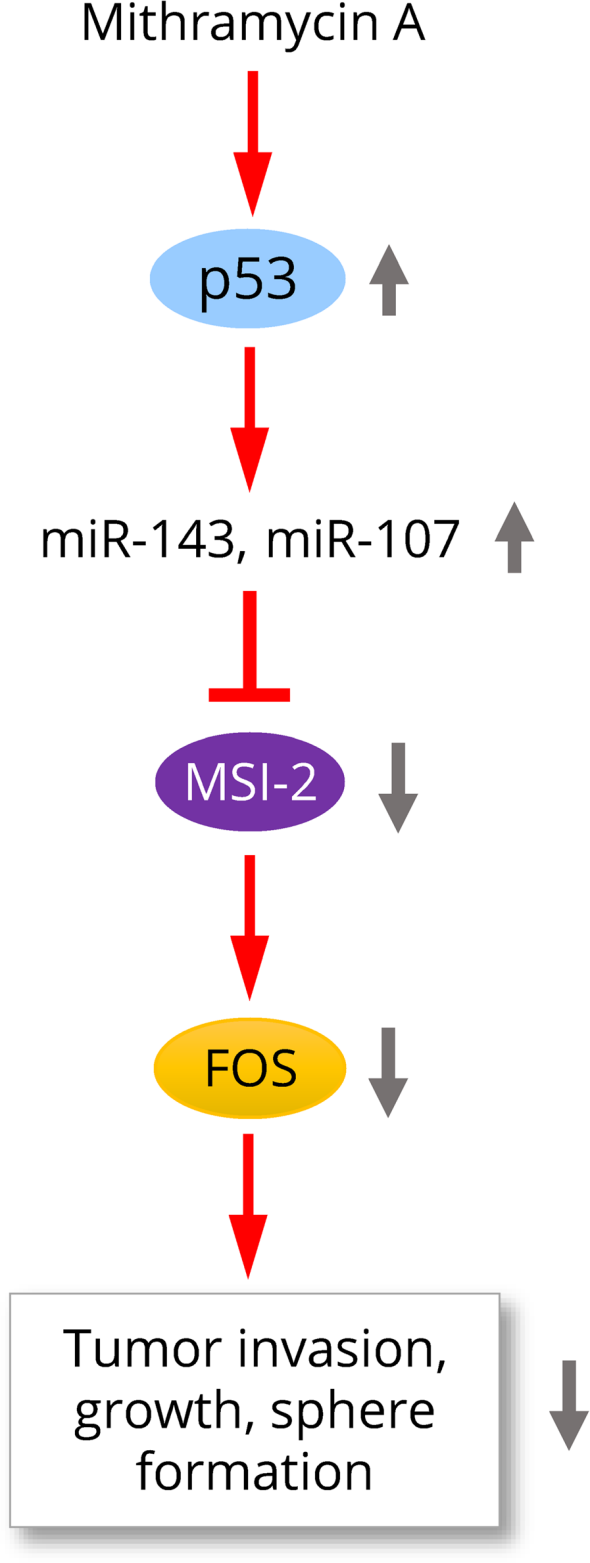
Schematic model depicting the role of the p53-miR-143/miR-107-MSI-2 axis in cervical cancer progression. Overexpression of MSI-2 upregulates the expression of c-FOS, which in turn promotes CC cell invasion, proliferation and sphere formation. Treatment with Mithramycin A restored the expression of miR-143 and miR-107 (two direct suppressors of MSI-2) via activation of p53, leading to inhibition of MSI-2 expression and reduced proliferation, invasion and sphere formation of CC cells

In summary, our study has revealed novel roles of MSI-2-c-FOS axis during CC progression, and the restoration of tumor suppressors miR-143 and miR-107 by Mithramycin A via activation of p53 could be an effective approach for the downregulation of MSI-2, resulting in the inhibition of invasion and metastasis (Fig. 7).

## Abbreviations

CC: cervical cancer
miRNAs: microRNAs
MM: Mithramycin A
MSI-2: Musashi-2
